# Fatty Acids and Oxylipins in Osteoarthritis and Rheumatoid Arthritis—a Complex Field with Significant Potential for Future Treatments

**DOI:** 10.1007/s11926-021-01007-9

**Published:** 2021-04-28

**Authors:** Anne-Mari Mustonen, Petteri Nieminen

**Affiliations:** 1 Institute of Biomedicine, School of Medicine, Faculty of Health Sciences, University of Eastern Finland, P.O. Box 1627, FI-70211 Kuopio, Finland; 2 Department of Environmental and Biological Sciences, Faculty of Science and Forestry, University of Eastern Finland, P.O. Box 111, FI-80101 Joensuu, Finland

**Keywords:** Fatty acid, Osteoarthritis, Oxylipins, Polyunsaturated fatty acids, Rheumatoid arthritis, Specialized pro-resolving lipid mediators

## Abstract

**Purpose of Review:**

Osteoarthritis (OA) and rheumatoid arthritis (RA) are characterized by abnormal lipid metabolism manifested as altered fatty acid (FA) profiles of synovial fluid and tissues and in the way dietary FA supplements can influence the symptoms of especially RA. In addition to classic eicosanoids, the potential roles of polyunsaturated FA (PUFA)-derived specialized pro-resolving lipid mediators (SPM) have become the focus of intensive research. Here, we summarize the current state of knowledge of the roles of FA and oxylipins in the degradation or protection of synovial joints.

**Recent Findings:**

There exists discordance between the large body of literature from cell culture and animal experiments on the adverse and beneficial effects of individual FA and the lack of effective treatments for joint destruction in OA and RA patients. Saturated 16:0 and 18:0 induce mostly deleterious effects, while long-chain n-3 PUFA, especially 20:5n-3, have positive influence on joint health. The situation can be more complex for n-6 PUFA, such as 18:2n-6, 20:4n-6, and its derivative prostaglandin E_2_, with a combination of potentially adverse and beneficial effects. SPM analogs have future potential as analgesics for arthritic pain.

**Summary:**

Alterations in FA profiles and their potential implications in SPM production may affect joint lubrication, synovial inflammation, pannus formation, as well as cartilage and bone degradation and contribute to the pathogeneses of inflammatory joint diseases. Further research directions include high-quality randomized controlled trials on dietary FA supplements and investigations on the significance of lipid composition of microvesicle membrane and cargo in joint diseases.

## Introduction

### Joint Diseases and Fatty Acids

Osteoarthritis (OA) and autoimmune-driven rheumatoid arthritis (RA) are chronic joint diseases characterized with inflammation, cartilage destruction, joint pain, and functional limitations eventually leading to disability [1, 2]. Other hallmarks include the development of osteophytes in OA and the production of autoantibodies, pannus formation, and bone erosion in RA. Obesity is a known risk factor for OA, which is featured with a lower inflammatory load (cells and cytokines in synovial fluid (SF)) compared to RA, and the enzymatic pathways involved in inflammation and the resolution of inflammation may be less activated in OA [3]. Anti-inflammatory and immunosuppressive medication can offer symptomatic relief but not reverse the cartilage damage that has occurred due to OA or RA [4, 5]. Potential biomarkers are actively investigated to enable diagnosing these diseases as early as possible to prevent irreversible joint damage and subsequent disability [6].

Both OA and RA are characterized by altered lipid metabolism, and lipids have been proposed to play several roles in their pathogeneses [7–9]. Increased body fat mass is a risk factor for OA development, as it induces mechanical stress, especially in the tibiofemoral joint, and causes systemic low-grade inflammation [10, 11]. Obesity can also be characterized with increased free fatty acid (FA) levels in circulation [12] and with increased ratios of n-6 polyunsaturated FA (PUFA) to n-3 PUFA both in the diet and in the body [13]. PUFA can induce favorable or detrimental effects on joint tissues, partly through their downstream derivatives, oxylipins. Other structural categories of FA in the diet and body with potential influence on joint pathology include saturated FA (SFA) and monounsaturated FA (MUFA). The nomenclature of FA is based on carbon chain length and the number and location of double bonds. SFA lack double bonds, whereas MUFA and PUFA have one and two or more double bonds, respectively. The most abundant and biologically important FA discussed in this review include 16:0 (palmitic acid), 18:0 (stearic acid), 18:1n-9 (oleic acid), 18:2n-6 (linoleic acid), 18:3n-3 (*α*-linolenic acid), 20:4n-6 (arachidonic acid), 20:5n-3 (eicosapentaenoic acid), and 22:6n-3 (docosahexaenoic acid). In general, FA bind to cellular membrane-bound or nucleus-located targets and induce the transduction of transmembrane or nucleus-specific signals [14]. This causes modulation of target gene transcription and protein synthesis and contributes to the regulation of cell growth, behavior, and function. Different cell types in synovial joints express receptors for FA (e.g., G protein-coupled receptors, Toll-like receptors, and peroxisome proliferator-activated receptors) and can use FA as an energy source [15].

**Fig. 1:**
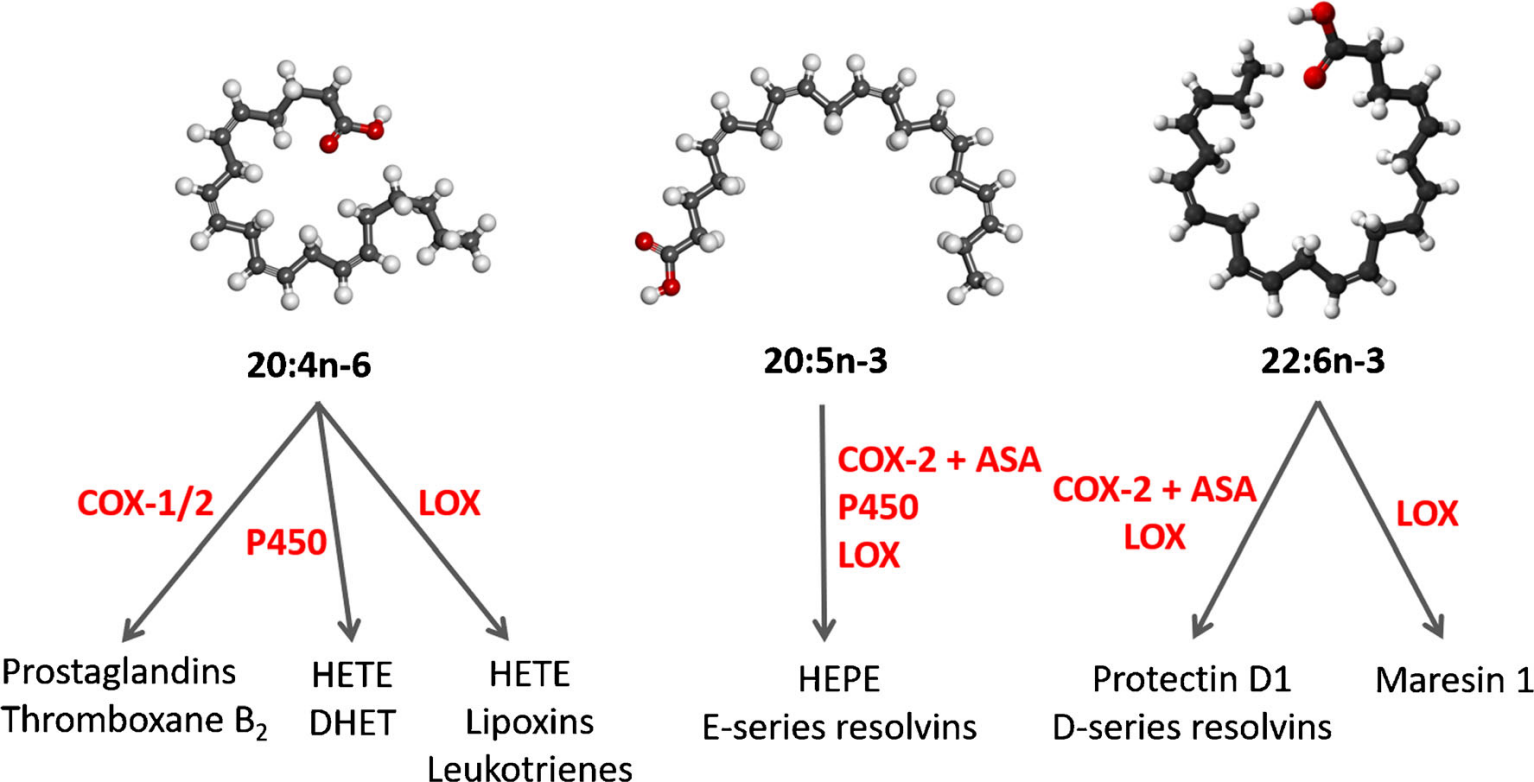
Overview of metabolic routes of 20C-polyunsaturated fatty acid-derived oxylipin discussed in this review [based on 20, 22, 155]. Note that not all metabolites are shown. ASA, aspirin; COX, cyclooxygenase; DHET, dihydroxyeicosatrienoic acid; HEPE, hydroxyeicosapentaenoic acid; HETE, hydroxyeicosatetraenoic acid; LOX, lipoxygenase; P450, cytochrome P450. Open source images provided by Creative Commons, (https://commons.wikimedia.org/wiki/File:Arachidonic_acid2.png, https://commons.wikimedia.org/wiki/File:Eicosapentaenoic_acid2.png, https://commons.wikimedia.org/wiki/File:Docosahexaenoic-acid-3D-balls.png)

PUFA-derived oxylipins include classic eicosanoids (prostaglandins, thromboxanes, leukotrienes) and specialized pro-resolving lipid mediators (SPM: lipoxins, resolvins, protectins, maresins) [16] (Fig. 1). They provide a link between FA and inflammation in synovial joints. For example, when synoviocytes are stimulated with interleukin (IL)-1*β*, the pro-inflammatory PUFA 20:4n-6 is released from biomembrane phospholipids (PL) after their hydrolysis by phospholipase A_2_ [17], the activity of which is high in OA and RA SF [18]. This leads to increased production of the inflammatory mediator prostaglandin E_2_ (PGE_2_) from 20:4n-6 by synoviocytes [17]. PGE_2_ and nitric oxide (NO) play important roles in the pathogenesis of OA together with cartilage-degrading enzymes [19]. The most important of these proteinases consist of two enzyme families, matrix metalloproteinases (MMP) and a disintegrin and metalloproteinase with thrombospondin motifs (ADAMTS) [15]. In addition to pro-inflammatory lipid mediators, several SPM are present in diseased joints [3, 20]. Normally, the inflammatory response is self-limiting, but it can become chronic in pathological conditions, such as RA, in which inflammation fails to resolve [21]. N-3 PUFA and oxylipins derived from them have often been described as anti-inflammatory, whereas n-6 PUFA and their derivatives have been considered pro-inflammatory. In vivo, the situation is not as simple, and the interactions of n-3 and n-6 PUFA in inflammation are still not properly understood [22]. For instance, the pro-resolving lipoxin A_4_ (LXA_4_) and pro-inflammatory prostaglandins are synthesized from the same precursor (20:4n-6).

### Aim of the Review

Our aim is to review the key literature on the manifestations and possible roles of FA in the development of OA and RA. We shall consider the sources of FA in the joint space including circulating lipids and the potential impact of adjacent tissues, especially the infrapatellar fat pad (IFP) of the knee, on joint diseases, the complexity of pro- and anti-inflammatory effects induced by FA and their derivatives, and the eventual possibility of therapeutic interventions.

## Literature Search

PubMed and Web of Science literature search was performed with the following keywords: “fatty acid,” “osteoarthritis,” “rheumatoid arthritis,” “chondrocyte,” “cartilage,” “bone,” “synoviocyte,” “synovium,” and “infrapatellar fat pad.” The search was restricted to articles published in full in English up to February 2020. One author (A-MM) screened the titles and abstracts of potential literature and determined their eligibility. Review articles were also included, and the bibliographies of relevant articles were examined for additional references. A total of 171 papers were incorporated in this review. Due to the vast amount of literature available, many relevant and overlapping papers were excluded to control the amount of references. In addition, papers on the shortest-chain FA were not included, even though it is known, for instance, that short-chain FA produced by gut microbiota can be involved in RA [23]. Thus, this review focuses on the effects of 12–24C FA on joint health with special emphasis on OA and RA.

## Osteoarthritis

### Dietary Fatty Acids and Osteoarthritis

The food FA composition is known to have a direct influence on the FA profiles of the body [13]. For instance, dietary n-3 PUFA administration replaces n-6 PUFA, particularly 20:4n-6, from the cellular membrane PL where 20:4n-6 is a major PUFA constituent [13, 22]. The effects of dietary FA intake on joint health have been mostly studied in laboratory rodents, humans, and horses, the knee being the most frequently investigated joint. The obtained data are in this case relatively uniform. The higher consumption of SFA or n-6 PUFA has been shown to be associated with synovitis, cartilage degradation, and progression of OA [24, 25]. Regarding individual SFA, 12:0 and 14:0 can have protective influence on joints compared to 16:0 and 18:0, which increase cartilage destruction, subchondral bone changes, and pain perception in rodent models of OA [26, 27]. Neither should all n-6 PUFA be considered detrimental [28]. For example, conjugated linoleic acid (CLA) that is a group of 18:2n-6 isomers (e.g., *cis*-9, *trans*-11 CLA) may reduce cartilage degradation and increase its regeneration in horses [29].

N-3 PUFA generally possess anti-inflammatory effects that are partly conveyed through the replacement of 20:4n-6 from membrane PL, as well as via the competitive inhibition of 20:4n-6 metabolism [13, 22]. Regarding individual n-3 PUFA, a distinction should be made between 18:3n-3 and its longer-chain derivatives. The potential anti-inflammatory effects of dietary FA can be small, if they mostly contain 18:3n-3 from plant sources, as its conversion to longer-chain n-3 PUFA is slow [13]. The influences of 20:5n-3 and 22:6n-3, found primarily in marine oils, are usually the opposite to SFA and n-6 PUFA: reduced OA pathology in cartilage and subchondral bone, decreased discomfort and lameness, pain alleviation, and better joint function [24, 30–32]. Delayed incidence of surgically induced knee OA was documented in *fat-1* transgenic mouse [33]. While there is no endogenous interconversion between the n-6 and n-3 PUFA categories in wild-type mammals [13], this experimental animal model converts dietary n-6 PUFA to n-3 PUFA resulting in considerable amounts of 18–22C n-3 PUFA and a decreased n-6/n-3 PUFA ratio in the body [34].

However, the situation is not totally straightforward, and partly conflicting results regarding n-3 PUFA have also been reported. Cai et al. [35] did not observe a delay in the development of idiopathic OA in *fat-1* mouse strain compared to wild-type mice. Neither were the SF biomarkers of horses altered by long-chain n-3 PUFA supplementation during experimentally induced synovitis [36]. Even though the expression of an aggrecanase, ADAMTS4, decreased in synovium, the overall gene expression pattern in cartilage remained unresponsive. Moreover, in a mouse model of surgically induced OA, animals fed a high-fat diet supplemented with n-3 PUFA had elevated levels of serum PGE_2_ [24]. As a final example, the lower dose of dietary fish oil led to greater improvements of pain and function scores than the higher, anti-inflammatory dose in a randomized, double-blind trial on OA patients [37]. There were no differences in the loss of cartilage volume between the doses after 2 years.

The association of dietary MUFA with joint health has been investigated less intensely than the possible roles of SFA and PUFA. In a relatively large study on humans (*n* = 2,092), the higher self-reported amounts of MUFA and PUFA in the diet were associated with reduced radiographic progression of knee OA [25]. This was supported by another study on women (*n* = 200) with an inverse association between the self-reported dietary intakes of MUFA and PUFA and the prevalence of radiographic knee OA [38]. In the above-mentioned studies, the n-3 and n-6 series PUFA were not presented separately. In a large-scale study on postmenopausal women (*n* = 80,551), the self-reported dietary intakes of 20–22C n-3 or 18–20C n-6 PUFA showed no association with the risk of OA [39]. In all these cases, it must be kept in mind that self-reported diets can be subjective and prone to errors.

In summary, the consumption of SFA and n-6 PUFA could be associated with increased risk of developing OA, whereas n-3 PUFA and MUFA may induce the opposite effects. A dietary n-6/n-3 PUFA ratio of 1–5:1 has been suggested to be suitable for the prevention and treatment of OA [40]. However, the evidence for a role of dietary PUFA in the improvement of OA pain and function is not robust in human patients [41]. Even though the studies on animal models have been promising, it has not yet been possible to prevent the progression of OA in humans by dietary means.

### Circulating Fatty Acids in Osteoarthritis

Dietary FA are mainly transported in FA–albumin complexes or in lipoproteins in plasma [15]. Currently, there is a lack of biomarkers to predict and monitor OA progression, and, due to the ease of sampling, it would be most practical to analyze plasma/serum or urine samples in search for potential lipid biomarkers in joint diseases. According to Bruderlein et al. [42], OA patients did not have altered FA composition in serum PL. In plasma, post-prandial SFA and PUFA levels were positively associated with clinically defined hand OA and with structurally defined knee OA, but only in men [43]. In addition, plasma n-3 PUFA levels in men were associated with hand OA and those of n-6 PUFA with structural knee OA and joint effusion. Hand or knee pain was not associated with these structural categories of FA. There also exist data that show no significant associations between circulating levels of total n-6 or n-3 PUFA and OA in humans [44].

In another study on humans, fasting plasma PL levels of 22:6n-3, but not those of 20:5n-3, were inversely associated with patellofemoral cartilage loss but were not associated with knee synovitis [45]. In addition, 20:4n-6 percentages showed a positive association with synovitis but not with cartilage loss. According to Sibille et al. [46], plasma n-6/n-3 PUFA ratios showed positive associations with pain symptoms and functional limitations of knee OA. Regarding rodent models, serum n-3 PUFA and n-3/n-6 PUFA ratios correlated inversely with OA severity, and 18:0 and several n-6 PUFA correlated positively with OA severity and/or synovitis [47]. In addition, the serum levels of 15:0, 16:1n-7, and 22:1n-9 correlated inversely with joint degradation and that of 24:1n-9 with synovitis.

Considering the derivatives of 20:4n-6, OA was associated with elevated levels of plasma PGE_2_ and 15-HETE (hydroxyeicosatetraenoic acid) in humans [48]. 15-HETE is a molecule with potential pro- and anti-inflammatory properties. In an experimentally induced synovitis model on horses, increased levels of several 20:4n-6-derivatives were documented in SF after lipopolysaccharide (LPS) challenge [49]. These included, for instance, PGE_2_, prostaglandin D_2_ (PGD_2_), thromboxane B_2_ (TXB_2_), leukotriene B_4_ (LTB_4_), and 5-, 11-, and 15-HETE. Some of the variables mentioned in this section, such as 15-HETE, could have future potential as biomarkers for the diagnosis and monitoring of early OA, and their synthesis paths as possible targets for treatment or prevention.

### Fatty Acids in Osteoarthritic Synovial Fluid

SF is an ultrafiltrate of plasma [7], and the SF FA profiles reflect circulating lipid levels [47]. The collection of SF is somewhat invasive and involves the risk of infection, which limits the collection of samples especially from healthy controls. In addition, a healthy knee often has only a small amount of SF that is difficult to remove. For ethical constraints, OA and RA SF are often compared to samples from *post-mortem* donors without joint diseases [8] or to patients with traumatized knees unrelated to OA/RA [50]. Still, cadaveric tissue does not represent an ideal control cohort, and SF lipid profiles are known to change due to trauma [51]. Information on the lipid profiles of healthy and diseased SF would be valuable due to the close physical association of SF with articular cartilage and synovium. Articular cartilage has a PL cover, which functions as boundary lubricant during joint loading together with other molecules, such as hyaluronic acid and lubricin [8, 52]. Changes in the FA composition of this PL layer can hypothetically affect lubrication efficiency, friction, and cartilage degradation. Generally, PL with longer-chain and more unsaturated FA are presumed to reduce the coefficient of friction in joints [53].

The SF proportions of 22:6n-3 and total n-6 PUFA were documented to decrease in end-stage OA together with increased percentages of 18:1n-9 and total MUFA in humans [50]. Late-/end-stage OA was also associated with reduced levels of 20:4n-6 and/or elevated levels of 10:0, 14–18C SFA, 16:1n-7, 18:1n-9, 18:3n-3, 20:4n-6, 24:0, and 24:1n-9 [54, 55]. N-6/n-3 PUFA ratios decreased in one study on human OA SF, but, in this case, the 18C PUFA precursors were not included in the calculation [55]. Controversy exists regarding the direction of change in the carbon chain lengths of the SF FA pool. Mustonen et al. [50] documented decreased average chain lengths of FA in the SF total lipids of OA patients. In contrast, Kosinska et al. [8, 52] noted increased FA chain lengths in phosphatidylcholine and phosphatidylethanolamine-based plasmalogen classes, but shorter-chain lengths in lysophosphatidylcholine fraction in OA. The significance of these mutually opposite findings remains to be determined.

The SF levels of C14–16 SFA, 16:1n-7, 18:3n-6, and most n-3 PUFA correlated inversely with OA severity in mice [47]. Animal models and humans have been characterized by altered 20:4n-6 metabolism that is reflected in OA SF [44, 56]. Lipid mediators derived from 20:4n-6 can be considered pro-inflammatory (e.g., PGE_2_, 8,9-dihydroxyeicosatrienoic acid (DHET)), anti-inflammatory (e.g., LXA_4_), or, in some cases, both (e.g., 15-HETE). In OA SF, 15-HETE was reported to decrease, while 8,9-DHET showed variable responses [44, 56]. The SF levels of resolvin D1 (RvD1), derived from 22:6n-3, were elevated in OA dogs [57]. de Visser et al. [56] suggested that oxylipins could play different roles in local (joints) and peripheral (circulation) compartments.

PL metabolism is altered during OA progression with several PL classes and species being elevated in OA SF [8]. The PL profile is of importance, as altered levels and composition of SF boundary lubricants may affect the scavenging of reactive oxygen species, inflammatory status, friction, and progression of cartilage damage. That said, the results on OA-induced changes in the SF profiles of individual FA do not always overlap, even though the levels of n-6 PUFA generally show a decreasing trend and those of long-chain MUFA may increase. Current literature displays a somewhat conflicting picture of the direction of inflammatory processes in the OA joint. This also applies to the data on circulating levels of these molecules. It is not known whether the observed changes are causes or consequences of OA, or both. Furthermore, when several dozens or even hundreds to thousands of variables are measured from the same samples, as is the case in some metabolomic and lipidomic studies, the probability for type I error increases. In such cases, it is not necessarily easy to reproduce the observed OA-related changes in subsequent experiments.

### Effects of Fatty Acids on Cartilage

Depending on the study, OA cartilage has been characterized with elevated levels of total FA and 20:4n-6 [58], 16:0, 18:0, 16:1n-7, 18:1n-9, and 18:2n-6 [59], or very-long-chain FA [60]. Among these, SFA 16:0 is the individual FA that is often included in the in vitro models of cartilage inflammation. This is understandable, as 16:0 has been shown to induce deleterious effects in different experimental models [61], but its use in isolation has also been criticized [62]. The in vitro effects induced by a single FA are not necessarily biologically relevant, as in vivo the tissue FA profiles are composed of a complex FA mixture. In the case of 16:0, its adverse effects are often cytotoxic and reversed by co-incubation with 18:1n-9.

In chondrocytes or cartilage explants of animal and human origin, 16:0 was documented to induce the expression of cyclooxygenase (COX)-2 and inducible NO synthase (iNOS), IL-6 release, endoplasmic reticulum stress, apoptosis, proteoglycan (PG)/glycosaminoglycan (GAG) loss, extracellular matrix (ECM) degradation, and cartilage breakdown [26, 63–66]. It can also produce some of these effects together with 18:1n-9 [67, 68]. However, once again the results are not totally consistent, as opposite data exist reporting reduced GAG release and cartilage breakdown [69] or no response to 16:0 exposure [70]. The stimulation of chondrocytes/cartilage with 18:0 induced the expression of cytokines and cartilage-degrading proteinases, loss of PG/GAG, as well as increased apoptosis [26, 65, 66, 71]. Shorter-chain SFA, 12:0 and 14:0, have also been studied in this respect, and they were associated with increased IL-6 secretion and GAG release [26, 72]. On the other hand, there exists literature showing less harmful effects of 12:0 and 14:0 on cartilage health compared to 16:0 and 18:0 [26, 66].

Regarding MUFA, chondrocytes and cartilage responded to exposure with 18:1n-9 by decreases in COX-2 expression, GAG release, and cartilage destruction [69], an increase in IL-6 secretion [72], or with no response [63, 70]. Lee et al. [65] observed increased apoptosis but only when 18:1n-9 was used in pathological concentrations. Co-incubation of 18:1n-9 and 16:0 reduced 16:0-induced apoptosis, and this was associated with accumulation of lipid droplets in articular cartilage. It was proposed that the 16:0-induced lipotoxicity could be prevented through the sequestration of excess free FA within these lipid droplets. In other studies, co-incubation of 16:0 and 18:1n-9 increased the production of IL-6, IL-8, and reactive oxygen species, as well as apoptosis [67, 68]. Once again, there remains some controversy further emphasizing the complex nature of lipidology in joint diseases.

Among n-6 PUFA, exposure of human OA chondrocytes to CLA caused reductions in PGE_2_ and NO production [73]. 18:2n-6 is the dietarily essential n-6 PUFA that is converted to 20:4n-6—the precursor of PGE_2_ that promotes inflammation in joint diseases [22]. 18:2n-6 has increased IL-6 secretion from human chondrocytes [72]. 20:4n-6 has been observed to accumulate in articular cartilage in proportion with the histological severity of OA [58]. Cultures of human OA or bovine chondrocytes supplemented with 18:2n-6 displayed increased PGE_2_ production [69], elevated NO but decreased PGE_2_ production [73], or no response [70], whereas 20:4n-6 increased COX-2 protein levels in bovine chondrocytes [74]. Co-incubation of human OA chondrocytes with 18:2n-6 and 20:4n-6 did not affect PGE_2_ production but suppressed that of NO [73]. Another study using bovine chondrocytes failed to detect any effects of 20:4n-6 on the expression levels of IL-1*α* or -*β*, tumor necrosis factor *α*, COX-2, ADAMTS4–5, MMP-3, or MMP-13 [75]. In canine chondrocytes, exposure to 20:4n-6 increased PGE production and ADAMTS5 expression [76]. Making the situation of the diverse outcomes more complex, the expression of MMP-3 was downregulated together with that of iNOS, resulting in reduced production of NO. Thus, 20:4n-6 can also have beneficial effects on cartilage in a cellular model of canine OA. Furthermore, PGE_2_ was shown to be important for maintaining cartilage homeostasis, in addition to its catabolic influence. At concentrations lower than those observed in inflammation, it became chondroprotective and suppressed the expression of pro-inflammatory cytokines and collagen degradation in OA cartilage [77].

In respect to n-3 PUFA, 20:5n-3 and 22:6n-3 have been utilized in research more often than the dietarily essential 18:3n-3 that, as stated above, is only rather slowly converted to its long-chain derivatives in the body [13]. There is ample evidence that 18:3n-3, 20:5n-3, and/or 22:6n-3 are able to reduce the expression of COX-2, iNOS, inflammatory cytokines, and cartilage-degrading proteinases and to decrease the production of PGE_2_, leading to reduced inflammation, chondrocyte apoptosis, GAG loss, and ECM degradation [70, 74–76, 78–81]. In several studies, 20:5n-3 was shown to be the most potent n-3 PUFA [75, 76, 78, 79], while 18:3n-3 was the least effective [75]. Intra-articular 20:5n-3 has prevented the progression of surgically induced OA by reducing chondrocyte apoptosis and MMP-13 expression in a mouse model [80]. Interestingly, 20:5n-3 and/or 22:6n-3 have also induced mild catabolic responses in the absence of IL-1*β*, with increased expression of cartilage-degrading proteinases [81] and loss of GAG [78]. In addition, dietary fish oil rich in 20:5n-3 and 22:6n-3 was noted to reduce 18:2n-6 and 20:4n-6 contents together with PG synthesis in the articular cartilage of rats [82].

To sum up, the in vitro effects of FA have been intensively investigated regarding articular cartilage, but the selection of FA used in these experiments has not been very diverse. The influence of selected SFA (16:0, 18:0) has mostly been deleterious, whereas long-chain n-3 PUFA, especially 20:5n-3, have shown opposite effects on the expression of markers of inflammation and cartilage degradation (Table 1). To the best of our knowledge, 20–24C MUFA have not been investigated in this respect. They could still be relevant in joint diseases, as OA is associated with peroxisomal dysfunction and the accumulation of very-long-chain FA in chondrocytes [60]. OA cartilage can contain elevated levels individual FA deemed unfavorable [58, 59], and it could be postulated that high SFA levels in obesity [83] would contribute to OA progression by increasing chondrocyte death. However, most in vitro studies suffer from the fact that they only use one FA at a time, which does not reflect the actual biological state within the joint and makes the extrapolation of the effects to whole organisms difficult.

**Table 1:** Potential in vitro and in vivo effects of individual fatty acids on joint tissues of animals and humans based on literature referenced in this review

	Effects on synovium	Effects on cartilage	Effects on bone
SFA			
12:0	–	+ –	+
14:0	–	+ –	+
16:0	–	– +	–
18:0	–	–	–
MUFA			
18:1n-9	–	+ –	+
n-6 PUFA			
18:2n-6	+ –	– +	–
CLA		+	+
20:4n-6	+ –	– +	+
n-3 PUFA			
18:3n-3	+	+	+
20:5n-3	+ –	+ –	+
22:6n-3	+	+ –	+

*SFA* saturated fatty acid, *MUFA* monounsaturated fatty acid, *PUFA* polyunsaturated fatty acid, *CLA* conjugated linoleic acid

+ = beneficial effects on joint health, – = deleterious effect on joint health

### Osteoarthritic Synovium and Infrapatellar Fat Pad

When human OA fibroblast-like synoviocytes (FLS) were stimulated with SFA 16:0 with or without inflammatory conditions, the expression levels of COX-2 and IL-6 increased [63]. MUFA 18:1n-9 had no significant effects on the same variables. Frommer et al. [72] documented 16:0- and 18:2n-6-induced secretion of IL-6 from OA synovial fibroblasts, but there was variation in responses between cells from different patients. In contrast, equine synovial fibroblasts incubated with 20:5n-3 or 22:6n-3 prior to the exposure to recombinant IL-1*β* had decreased expression levels of COX-2, IL-1*β*, IL-6, MMP-1, MMP-13, and ADAMTS4 [84]. In addition, 22:6n-3 increased the synthesis of RvD1–2, maresins MaR1–2, and protectin DX (PDX), all classified as SPM, by synoviocytes. 18:3n-3 reduced LPS-induced production of PGE_2_ from synovial membrane explants of horses [85]. On the other hand, the stimulation of equine synoviocytes with 18:2n-6 did not affect the gene expression patterns of COX-2, IL-6, or proteinases, but reduced the expression of IL-1*β* in a manner comparable to 20:5n-3 and 22:6n-3 [84]. Regarding eicosanoids, most OA joints have calcium-containing crystals that have been demonstrated to increase PGE_2_ production by fibroblasts via COX-1–2 [86]. As PGE_2_ mediates IL-1*β*-induced cartilage destruction, the inhibition of its synthesis is the cornerstone of the pharmacologic treatment of OA. However, PGE_2_ may also reduce IL-1*β*-induced inflammatory cell recruitment in synovium and can, thus, induce beneficial effects in OA. LXA_4_ from 20:4n-6 suppresses the synthesis of IL-6, IL-8, and MMP-3 with a parallel increase of tissue inhibitor of metalloproteinases TIMP-1 and TIMP-2 in synovial fibroblasts [87].

IFP or Hoffa᾽s fat pad has recently been studied for a role in OA, as there is possible cross-talk between this intracapsular but extrasynovial organ and articular tissues in the knee joint [88]. Adipocytes and infiltrating immune cells in IFP are potential sources of several inflammatory mediators including cytokines, chemokines, and adipokines, such as leptin and adiponectin [89, 90]. In OA, IFP was documented to display a pro-inflammatory phenotype [89]. It has been proposed to contribute to the pathophysiological processes in the knee joint by secreting FA and oxylipins [91]. The role of IFP in OA has been studied by stimulating FLS from end-stage disease with IFP-conditioned medium resulting in increased expression and/or secretion of COX-2, PGE_2_, IL-6 and IL-8, and cartilage matrix-degrading MMP-1, MMP-3, MMP-9, and MMP-13 [92]. These data suggest pro-inflammatory and catabolic roles for IFP in the end-stage OA. Other prostaglandins that IFP has been documented to secrete include, for instance, PGD_2_, PGD_3_, and prostaglandin F_2α_ (PGF_2α_) [93], the last of which induces increased collagen production (fibrosis) in OA FLS [94].

In humans, end-stage OA was associated with increased secretion of 20:4n-6, 22:6n-3, and TXB_2_ from IFP to fat-conditioned medium, while the secretion of 20:4n-6-derived anti-inflammatory LXA_4_ was lower compared to *post-mortem* donors [93]. It was also noted that increases in the levels of individual FA were not necessarily associated with increased levels of oxylipins derived from the same FA. Neither were the FA proportions in SF and IFP directly interrelated in human subjects [50]. Elevated IFP proportions of 20:4n-6 and 22:6n-3 together with lowered total MUFA percentages and n-3/n-6 PUFA ratios in a rabbit model of early OA [95] partly confirmed the secretion data from humans [93]. Van de Vyver et al. [55] suggested that inflammation could be less pronounced in the IFP of end-stage OA and this may be associated with the reduced n-6 PUFA levels in SF. This is supported by the findings of Bastiaansen-Jenniskens et al. [96], who stimulated bovine cartilage explants with fat-conditioned medium from the IFP of end-stage OA. They reported decreased release of NO and GAG and reduced expression of MMP-1 and MMP-3, indicating protective actions of IFP on cartilage damage. However, these results are contradictory to the data of Eymard et al. [92] discussed earlier, leaving the role of IFP in OA progression unresolved. Also in this case, there is a lack of control samples, as IFP is not routinely removed during arthroscopy unlike in total joint replacement surgery due to OA/RA [50].

### Effects of Fatty Acids on Bone

Subchondral bone underlies articular cartilage in synovial joints and is involved in OA pathology [15]. In cancellous bone with marrow, the levels of 20–22C n-6 PUFA, especially that of 20:4n-6, were elevated in OA bone compared to osteoporotic bone [97]. The proportion of 16:1n-7 was also higher, while that of 18:0 was lower in OA. In another study by Humphries et al. [98], both n-6 and n-3 PUFA proportions were higher in cancellous subchondral bone from OA patients compared to autopsy controls. 16:0 and 18:2n-6 can stimulate the secretion of IL-6 and chemokines from human OA osteoblasts [99]. As previously reviewed [14, 15], 16:1n-7, 18:1n-9, 18–22C n-3 PUFA, and 20:4n-6 can have anabolic effects on bone by promoting its formation and/or inhibiting resorption. Especially 20:5n-3 and 22:6n-3 stimulate osteoblastogenesis, survival, and activity of osteoblasts. In addition, they inhibit differentiation, proliferation, and maturation of osteoclasts and trigger their apoptosis. SFA 16:0, on the other hand, shows the opposite effects by suppressing osteoblast function, by increasing the apoptosis of osteoblasts and osteocytes, as well as by enhancing osteoclastogenesis. Among MUFA, 16:1n-7 is able to induce osteoclast apoptosis and to decrease bone resorption, while 18:1n-9 may prevent 16:0-induced lipotoxicity in osteoblasts. The roles that 20:4n-6 plays in bone are complex and still not properly understood.

As n-3 PUFA can have anabolic effects on bone formation and density [14, 15], they could theoretically promote OA by stimulating osteophytosis and subchondral bone formation. In contrast, dietary n-3 PUFA tended to decrease subchondral bone deposition in OA-prone guinea pigs [30]. Moreover, the reduced n-6/n-3 PUFA ratio in the body of transgenic *fat-1* mice did not influence the mineral density of subchondral cortical or trabecular bone [35]. On the other hand, rats fed with 16:0- or 18:0-rich diets had decreased subchondral bone density compared to those fed with 12:0 or 14:0 [26]. In a randomized controlled trial, low or high doses of dietary 20:5n-3 and 22:6n-3 in fish oil for 2 years had no effects on bone loss in knee OA patients [100]. PGE_2_ has been documented to exert complex influence on bone [14], while the anti-inflammatory LXA_4_ from 20:4n-6 could have preventive effects on bone loss [101].

All literature reviewed above does not derive from OA bone but is obtained from other research models. For this reason, potential associations between FA and arthritic manifestations of bone remain indefinite and need additional research to be clarified.

### Oxylipins in Osteoarthritic Joints

SPM include lipoxins derived from 20:4n-6 and resolvins, protectins, and maresins from 20:5n-3 and 22:6n-3 [16]. In addition to pro-inflammatory cascades, OA joints have been shown to contain these pro-resolving oxylipins and their precursors. For instance, 18-HEPE (hydroxyeicosapentaenoic acid), 17-HDHA (hydroxydocosahexaenoic acid), and RvD2 were detected from the SF of OA patients [3], and RvD1 was elevated in OA SF in dogs [57]. Potentially pro-inflammatory lipid mediators, such as 5-HETE, are also present in the SF of OA patients [3, 20]. 8,9-DHET, 11,12-DHET, and 14,15-DHET in SF were documented to be associated with radiographic progression of OA [44]. The deletion of 12/15-lipoxygenase (LOX) accelerated cartilage destruction via increased expression of MMP-13, ADAMTS5, and iNOS in aging-associated and surgically induced OA in mice [102]. The potentially anti-inflammatory metabolites of 12/15-LOX (15-HETE, 13-HODE (hydroxyoctadecadienoic acid), and LXA_4_) showed chondroprotective properties by decreasing the production of PGE_2_, NO, and MMP-13 in cartilage explants.

Exposure of human OA chondrocytes to RvD1 suppressed the expression of COX-2, iNOS, and MMP-13, decreased the release of PGE_2_ and NO, and reduced oxidative stress and apoptosis [57]. The expression levels of COX-2, IL-6, MMP-1, MMP-13, and ADAMTS4 decreased in equine synovial fibroblasts in response to RvD1–2, MaR1, and/or PDX [84]. In human macrophages, RvD1 suppressed pro-inflammatory markers and, in high-fat-fed mice, intra-articular RvD1 reduced the severity of surgically induced knee OA [103]. In addition to long-chain n-3 PUFA-derived SPM, anti-inflammatory lipid mediators are also derived from 20:4n-6. For instance, 15d-PGJ_2_ (15-deoxy-∆^12,14^-prostaglandin J_2_) reduced IL-6 synthesis in chondrocytes [104] and induced anti-fibrotic effects on OA synovial fibroblasts [105]. Moreover, intraperitoneal administration of LXA_4_ attenuated the progression of surgically induced OA in mice [102].

Inflammatory pain is usually treated with nonsteroidal anti-inflammatory drugs (NSAID), COX-2 inhibitors, and eventually also opiates in OA patients [106], but SPM may have future potential as analgesics. Exogenous administration of a D-series resolvin precursor 17-HDHA increased plasma RvD2 levels and reversed pain behavior in rat models of chemically and surgically induced OA [107]. Cartilage degeneration and synovitis in the knee joint were not affected, and the effects on pain perception were not subject to tolerance. In humans, the circulating levels of 17-HDHA, but not those of the studied resolvins, were associated with increased heat pain thresholds and lower intensity of chronic OA pain [108]. These effects were independent of 22:6n-3 levels and probably not induced by D-series resolvins.

To conclude, OA SF is known to contain a vast array of PUFA-derived lipid mediators that can potentially have either positive or negative influence on joint health. However, the combined effects of these multiple, simultaneously affecting compounds are difficult to assess. In addition to the COX/PGE_2_ pathway, the LOX pathway could be activated in OA [16]. The reviewed FA and oxylipin literature suggests that, in addition to destructive processes, there could be several compensatory mechanisms present and activated in the diseased joint to prevent further cartilage damage in OA. However, the SPM present are not able to resolve the inflammation in the diseased joint. They have been shown to be associated with pain in animal models and humans and could offer promising targets for translational research on pain relief in joint diseases.

## Rheumatoid Arthritis

### Effects of Dietary Fatty Acids on Rheumatoid Arthritis

So far, the potential effects of SFA on joint health have not been a focus of RA research. Consumption of dietary MUFA and especially olive oil, consisting mainly of 18:1n-9, may participate in the prevention of RA and suppress its disease activity [109, 110]. The ratio of dietary MUFA/SFA can correlate inversely with the disease activity score and erythrocyte sedimentation rate [110]. Compared to SFA and MUFA, the effects of long-chain n-3 PUFA have been more intensively investigated. In contrast to OA, in which the situation remains more complex and unresolved, 20:5n-3 and 22:6n-3 have been shown to induce beneficial effects on arthritic joints in both animal models [111–113] and RA patients [114–116]. Rodent models used to study RA pathogenesis often include collagen-induced arthritis, complete Freund᾽s adjuvant-induced arthritis, and K/BxN serum-transfer arthritis. It has been shown that n-3 PUFA in fish oil can be beneficial in rodent arthritis by reducing the production of pro-inflammatory cytokines and cartilage-degrading proteinases and by decreasing the migration of leukocytes [116]. In addition to reduced symptoms, pannus formation and cartilage and bone destruction could also be attenuated. 22:5n-3, an intermediary product between 20:5n-3 and 22:6n-3, can also resolve inflammation and reduce arthritis severity [117]. In a rat model of adjuvant-induced arthritis, dietary 22:6n-3 has increased the thickness of articular cartilage [118]. N-3 PUFA supplementation leads to an elevated local production of SPM, such as RvD1, and decreases in the levels of 20:4n-6-derived oxylipins in paws in serum-transfer-induced arthritis [113]. The transgenic *fat-1* mouse capable of endogenous conversion of n-6 PUFA to n-3 PUFA displays attenuation of arthritis induced by serum transfer [119].

In human patients, the influence of n-3 PUFA consumption is mostly manifested as decreased tender joint count, shorter duration of morning stiffness, and reduced pain [114–116]. The frequency of NSAID consumption can also decrease with n-3 PUFA supplementation. Usually the doses of 20:5n-3 and 22:6n-3 that relieve RA symptoms have varied between >2.7 and 6 g/day [114, 115]. On the other hand, a 3-month supplementation with 18:3n-3 was ineffective in RA patients [120]. The beneficial effects of long-chain n-3 PUFA could be caused by reduced numbers of inflammatory cells, decreased concentrations of pro-inflammatory mediators, as well as increased production of SPM at the site of inflammation. N-3 PUFA consumption has been inversely associated with the risk of developing RA [116, 121], and they could have potential in preventing the formation of autoantibodies in subjects at risk for RA [122]. N-3 PUFA may also induce other beneficial health effects, such as a reduction of cardiovascular risk [116, 123]. The influence of marine oils on synovial histopathology and radiographic progression of human RA remains mostly uninvestigated, but advantageous effects have been manifested in rodent models. For instance, krill oil with the majority of its n-3 PUFA in PL has decreased cell infiltrations, synovial hyperplasia, paw swelling, and clinical arthritis score in collagen-induced arthritis [111]. There remains some inconsistency regarding the effects of n-3 PUFA, as 22:6n-3 alone (1.0 or 2.5 g/kg/day) reduced arthritis severity in mice, while 22:6n-3 combined with 20:5n-3 (1.0 + 1.5 g/kg/day) lacked significant effects [112].

Regarding n-6 PUFA, dietary CLA reduced the manifestations of murine collagen-induced arthritis in a dose-dependent manner [124]. Beneficial effects were documented for clinical arthritic score, paw swelling, as well as cytokine levels, but the effective doses were too high (3 g *trans-*10, *cis-*12 CLA/day) to be realistically obtained from natural sources (dairy fat). 18:3n-6 consumption alone or in combination with n-3 PUFA was associated with decreased signs and symptoms of RA disease activity in humans [125, 126]. 18:3n-6 is elongated to 20:3n-6, the precursor of the anti-inflammatory PGE_1_ [22]. Even though 18:3n-6 and 20:3n-6 are also precursors for 20:4n-6, their anti-inflammatory potential may be of importance in RA [126]. According to Adam et al. [127], there could be synergism between low dietary 20:4n-6 intake and fish oil supplementation regarding inflammation in RA patients. On the other hand, dietary 20:4n-6 (or 22:6n-3) did not affect paw PGE_2_ content or arthritis severity in rats with adjuvant-induced arthritis [128].

A large study on postmenopausal women (*n* = 80,551) did not report significant relationships between self-reported n-6 or n-3 PUFA consumption (18:3n-3 not included) and the risk of RA [39]. There were no associations of dietary n-6 PUFA with unacceptable pain or refractory pain, even though n-3 PUFA intake correlated inversely and n-6/n-3 PUFA ratios directly with these pain parameters [129]. N-3 or n-6 PUFA intakes were not associated with inflammatory pain or systemic inflammation. According to de Pablo et al. [130], the levels of 18:2n-6 in erythrocyte total lipids correlated inversely with the risk of developing RA, making the situation regarding n-6 PUFA once again more complex. The levels of other n-6 PUFA, n-3 PUFA, or n-3/n-6 PUFA ratios showed no association with the risk of RA. In a large dataset on women (*n* = 166,013), the consumption of fish or marine n-3 PUFA had no protective effect on the risk of RA [131]. Instead, fish intake was associated with increased RA risk among individuals diagnosed at the age >55 years.

In brief, long-chain n-3 PUFA can induce beneficial effects on RA symptoms according to several reviews. They provide promising treatment options for joint pain associated with RA and may be effective in reducing NSAID use. Furthermore, some studies have shown an inverse association between n-3 PUFA intake and RA risk, but there has not been protective influence in others. Beneficial effects on joint histopathology have been mostly demonstrated in rodent models of RA.

### Circulating Fatty Acids in Rheumatoid Arthritis

Elevated proportions or concentrations of 14:0, 16:0, 16:1n-7, 18:1n-7, 18:1n-9, 20:1n-9, 20:2n-6, 20:3n-6, 22:1n-9, and/or total SFA were documented in plasma or serum of RA patients in comparison to controls [42, 132–134]. In addition, RA was characterized by reduced percentages or concentrations of, for instance, 16:0, 16:1n-7, 18:0, 18:1n-9, 18:2n-6, 18:3n-3, 20:4n-6, 20:5n-3, 22:4n-6, 22:5n-3, 22:6n-3, 20–22C MUFA, 24:0, and/or total n-6 PUFA in circulation compared to controls [42, 132–135]. There was an inverse correlation between serum C-reactive protein and 18:2n-6 in phosphatidylcholine in RA patients [133].

Regarding plasma oxylipins, PGE_2_, PGF_2α_, LTB_4_, TXB_2_, 5-, 8-, 12-, and 15-HETE, and 9- and 13-HODE were elevated among others in a rat model of RA [136]. Other studies on rodents also documented a large array of 18:2n-6-, 20:4n-6-, 20:5n-3-, and 22:6n-3-derived oxylipins in plasma with altered concentrations in arthritis [137]. Oxylipins from 18:2n-6 were downregulated, while those from the other PUFA-precursors were upregulated. Oxidation products of 18:2n-6 and 20:4n-6 (9-HODE, 13-HODE, 5-HETE, 15-HETE) were reported to increase in plasma high- and low-density lipoproteins in patients with active RA [138]. SPM, such as RvD3, RvD4, and RvE3, reduced in the serum of RA patients [139], while pro-inflammatory eicosanoids, such as LTB_4_, showed increased levels [140]. The central role of LTB_4_ has been established in rodent models but not for human RA [5, 141].

To conclude, analyses of plasma and serum samples from patients and rodent models have revealed somewhat inconsistent manifestations of RA in circulating FA and oxylipins. Similar to OA, finding a reliable blood biomarker for diagnosis of early RA or a target for its prevention would be of utmost importance but this has proven to be challenging.

### Fatty Acids in Rheumatoid Arthritis Synovial Fluid

The SF FA and oxylipin profiles are altered in several ways in RA, but the interpretation of these data is not always simple. The pre-analytical processing of the samples can differ between studies and different lipid fractions may be investigated, leading to confounding results. In many human studies, the results of RA patients were compared to those with OA [134] or to autopsy controls [8], due to the difficulty of obtaining healthy control samples. In general, SF from RA patients contains more total PL, major PL classes, and PL species compared to *post-mortem* donors [8], and these changes can potentially influence the FA profiles of SF.

RA SF was characterized with elevated percentages of 16:0, total SFA, long-chain MUFA, and/or total MUFA compared to SF from trauma controls or OA patients [50, 134]. In comparison to non-RA inflammatory arthritis, RA patients had lower levels of 14:0 and 16:1n-7 [142]. Among PUFA, decreased percentages of 18:2n-6, 18:3n-3, 20:5n-3, and/or total n-3 PUFA have been reported in RA compared to trauma control or OA SF [50, 134]. Elevated long-chain MUFA proportions may be associated with RA-induced increases in SF sphingomyelin levels [8]. Controversial results exist on the effects of RA on the chain length of SF FA, when measured from different lipid fractions after dissimilar pre-analytical processing of the samples [8, 50, 52].

The SF levels of PGE_2_, LTB_4_, and 15-HETE have been documented to be elevated in RA [19, 138, 140]. The case of PGE_2_ is interesting, as it has been suggested to play a dual role in arthritis [143]. In the inflamed phase, PGE_2_ would be pro-inflammatory, but it could also contribute to the resolution of inflammation by modulating the biosynthesis of LXA_4_. This may be associated with the perpetuating influence of COX-2 inhibitors on inflammation. In addition to LXA_4_, RA SF has been reported to contain other SPM, such as MaR1, RvD1, RvD3, and RvD5 [113, 144], or opposite to this, not to contain detectable amounts of SPM [3]. According to Sano et al. [20], most 20:4n-6-, 20:5n-3-, and 22:6n-3-derived metabolites from the COX, 5-LOX, 12/15-LOX, and cytochrome P450 pathways were elevated in RA SF compared to OA SF, and severe RA and severe OA could be differentiated by biomarkers, such as 5-HETE, 12-HETE, LXA_4_, protectin D1 (PD1), 12-HEPE, and 17-HDHA.

Some FA manifestations in SF, such as decreased proportions of essential 18C PUFA and 20:5n-3, can be similar to RA-related changes in circulation. The lowered n-3 PUFA levels could explain the good response of RA patients to marine oils. The literature on RA-related changes in oxylipin levels is also relatively variable ranging from no detectable SPM to elevated levels of particular SPM due to RA, but there is diagnostic potential regarding these molecules.

### Rheumatoid Arthritis, Synovial Tissue, and Infrapatellar Fat

Compared to OA FLS, the levels of 12–22C SFA, 16:1n-7, 18:1n-9, and 18:2n-6 were higher in RA FLS [145]. Regarding IFP, the proportions of 20:4n-6, 22:6n-3, and total n-6 PUFA were lower in RA compared to OA patients [50]. However, it remains to be verified, whether IFP releases considerable amounts of FA and oxylipins into SF. When human RA synovial fibroblasts were stimulated with 16:0 or 18:2n-6, the secretion of IL-6, chemokines, pro-MMP-1, and MMP-3 increased [72]. Several other FA, such as 12:0, 14:0, 16:1n-7, 18:0, 18:1n-9, and 20:5n-3, were also able to induce IL-6 secretion with no differences in the efficiencies between FA. The expressions of IL-6, MMP-3, and MMP-13 were documented to be suppressed in FLS from *fat-1* mice compared to wild-type mice [119].

An imbalance between FLS proliferation and apoptosis leads to pannus formation characterized by the aggressive and invasive behavior of FLS [146]. 20:4n-6 is able to enhance the proliferation of synovial cells [147]. Not surprisingly, high PGE_2_ levels in SF can contribute to RA synovial hyperplasia and pannus formation [146], whereas 20:5n-3 is able to suppress the proliferation in a dose-dependent manner [148]. Synoviocytes are capable of lipid mediator synthesis [3] and metabolize, for instance, exogenous leukotriene A_4_ to LTB_4_, which modulates synovial inflammation, FLS migration and invasion, and promotes joint erosion by the way of pannus formation [141]. 5-LOX, 15-LOX-1, and LTB_4_ levels were elevated in RA synovial tissue [140, 149], and COX-2 and PGE_2_ were increased in RA FLS [146]. PGE_2_ can induce both deleterious and beneficial effects on synovial fibroblasts, as it has also been demonstrated to prevent the overgrowth of synovial tissue in RA [150]. 15d-PGJ_2_ triggers synoviocyte apoptosis, inhibits pannus formation, as well as suppresses adjuvant-induced arthritis in rats [151]. Regarding cartilage-degrading enzymes, PGE_1–2_ are able to reduce MMP-1 release from FLS [152], and LXA_4_ may suppress cartilage destruction by reducing the synthesis of MMP-3 while increasing the production of TIMP-1–2 in synovial fibroblasts [87]. de Molon et al. [153] studied the effects of 20:5n-3-derived RvE1 on human RA synovial tissue but found no influence on the release of pro-inflammatory cytokines.

In brief, the exposure of synovial fibroblasts to individual FA affects the secretion of IL-6, but the potential roles of different FA categories remain indefinite. The COX/PGE_2_ and 5-LOX/LTB_4_ axes are activated in RA synovial tissue, and PGE_2_ may have a dual role in pannus formation.

### Effects of Fatty Acids on Cartilage and Bone

Some findings discussed in the OA section of this review are also relevant regarding RA. These include, for instance, the in vitro investigations that exposed normal chondrocytes to different FA under inflammatory conditions and demonstrated that 16:0 could exert pro-apoptotic and pro-inflammatory effects [63], while n-3 PUFA reduced the expression of cytokines and cartilage-degrading proteinases [75]. In a rat model of adjuvant-induced arthritis, a lowered 18:2n-6/18:3n-3 ratio in the diet decreased cartilage damage [40], and dietary 22:6n-3 increased articular cartilage thickness and reduced MMP-13 levels [118]. The former effect cannot be attributed to long-chain n-3 PUFA intake, as the diets were not reported to contain either 20:5n-3 or 22:6n-3 in significant amounts. In a mouse model of collagen-induced arthritis, dietary supplementation with 22:6n-3 decreased cartilage damage [112]. 20:4n-6 metabolites from synovial tissue may inhibit DNA synthesis in human articular chondrocytes and synovial fibroblasts as well as stimulate PG and collagen synthesis in articular chondrocytes [154]. The findings suggest that these compounds could play potential roles in the repair of damaged cartilage matrix and in the prevention of pannus formation. 17R-RvD1 (a stable epimer of RvD1) can stimulate the deposition of ECM in chondrocytes as well as protect K/BxN serum-challenged arthritic mice from cartilage degradation [113].

Regarding bone, the circulating levels of free FA can be elevated in RA and may have negative influence on the integrity of subchondral bone [99]. Exposure of human RA osteoblasts to 16:0 or 18:2n-6 has led to increased secretion of IL-6 and chemokines. The mineralization activity of osteoblasts correlated inversely with IL-6 secretion, suggesting a link between inflammation and reduced bone mineralization. FA did not directly affect osteoblastogenesis. In the same study, 16:0 and 18:2n-6 stimulated the secretion of IL-8 from human RA osteoclasts, while the other measured parameters, such as MMP-9, remained unaffected. There could be increased sensitivity to 16:0 and 18:2n-6 in RA compared to OA osteoclasts. RA can lead to an elevated risk to osteoporosis [155], and MUFA have been recognized as potential agents in the prevention of bone loss [14]. As reviewed by Bao et al. [14], 20:5n-3 and 22:6n-3 can increase bone formation, and 16:1n-7, 18:1n-9, 20:4n-6, and 22:6n-3 may suppress bone resorption, whereas 16:0 can exert opposite effects. Accordingly, osteoclastogenesis is reduced in the transgenic *fat-1* mouse [119]. In a murine model of collagen-induced arthritis, supplementation with 22:6n-3 decreased bone damage [112]. Similarly, dietary CLA may inhibit bone resorption and improve bone formation [156], while PGE_2_ is known to be one factor with bone-resorbing activity secreted by RA synovia [157]. RvD1 and RvE1 can reduce osteoclast differentiation and bone resorption in vitro [158, 159] and, in arthritic mice, RvD1 may decrease bone and cartilage destruction in vivo [159].

In summary, free FA have the potential to induce cartilage and bone damage, but more research is necessary, especially regarding their influence on subchondral bone. N-3 PUFA and their derivatives may have positive effects on cartilage and bone health in RA, while the situation could be more complex for n-6 PUFA with a combination of potentially beneficial and adverse effects (Table 1).

### Role of Extracellular Vesicles in Rheumatoid Arthritis

Virtually all cell types secrete extracellular vesicles (EV) into biological fluids, including SF [160]. They are nanosized membrane-coated particles, whose shedding is stimulated by diverse conditions, such as tissue regeneration and cancer progression. As EV release is also increased in inflammation, they could be expected to play a role in inflammatory and autoimmune diseases. Synovial fibroblasts were documented to secrete EV into SF [161], and their counts were altered by joint pathology as reviewed by Mustonen et al. [162]. Different EV types can be elevated in the plasma and SF of RA patients and relate to disease activity. EV could contribute to inflammatory processes and ECM degradation by transporting bioactive molecules and by stimulating FLS to produce inflammatory mediators and cartilage-degrading proteinases. For instance, 20:4n-6 can be transported by EV from leukocytes and to be subsequently converted to PGE_2_ by synovial fibroblasts [163]. In addition to FA, EV have the ability to transport eicosanoids, SPM, and their synthesis machinery [164, 165]. Transcellular synthesis of bioactive oxylipins may exert potentially deleterious or beneficial influence on RA tissues in chronic inflammation, and the same phenomena could also be applied to OA. Norling et al. [166] constructed nanoparticles that contained aspirin-triggered RvD1 or LXA_4_ analog, which were able to dampen the inflammatory responses in the mouse peritonitis and temporomandibular joint inflammation models. Based on these multi-faceted actions, it is not surprising that the potential of EV in the treatment of joint diseases is actively studied. It has been demonstrated, for example, that when exposing rats to osteochondral defects on distal femurs, a 12-week course of intra-articularly applied exosomes from human embryonic mesenchymal stem cells induced restoration of cartilage and subchondral bone [167].

To sum up, the potential roles of EV have become the focus of intensive research also regarding joint diseases, such as RA. EV have the ability to transport FA, oxylipins, and related enzymes as cargo to target cells and to modify the production of inflammatory mediators and cartilage-degrading enzymes in arthritic joints. Constructed nanoparticles with SPM may have therapeutic potential to treat joint inflammation in the future. However, the significance of lipid and FA composition of EV membrane is still only in its earliest stages of research and should be looked into in more detail, as FA have been shown to exert significant effects on joint health as evidenced by the large amount of material assessed in the present review.

### Oxylipins in Rheumatoid Arthritis Joints

SPM, such as MaR1, RvD5, and LXA_4_, are present in the SF of RA patients [144]. They could display anti-inflammatory actions in the diseased joint but are unable to resolve the inflammation associated with RA. It has been hypothesized that there might be under-functioning of SPM in RA, as macrophage metabolism fails to switch from a pro-inflammatory to a pro-resolving phenotype [168]. Compared to OA, the levels of 5-LOX and 15-LOX may be higher in RA, while the COX pathway could remain similar [3, 149]. However, it was recently documented that most 20:4n-6-, 20:5n-3-, and 22:6n-3-derived metabolites from the COX, 5-LOX, 12/15-LOX, and cytochrome P450 pathways could be elevated in RA SF compared to OA [20]. COX-2 is an integral part of the synthesis chain that not only drives inflammation via the production of PGE_2_, but also the resolution of inflammation via 15d-PGJ_2_ [143]. It has been hypothesized regarding some acute models that there could be a lipid mediator switch from PGE_2_ to 15d-PGJ_2_ during the transition from inflammation to resolution phase.

Resolvins could be beneficial in RA inflammation in several ways: (i) by reducing the recruitment of leukocytes, (ii) by decreasing the levels of pro-inflammatory mediators, (iii) by preserving bone, and (iv) by alleviating pain [155]. The levels of particular SPM (RvD1–3) were demonstrated to be elevated during the resolution phase of inflammation in K/BxN serum-challenged arthritic mouse [139]. Compared to self-resolving arthritic joints, the levels of RvD3, MaR1, and PD1 were lower and those of pro-inflammatory lipid mediators higher in delayed-resolving joints. RvD3 administrations decreased arthritis severity by reducing joint leukocyte numbers, eicosanoids levels, and paw edema. In another study on K/BxN serum-challenged mouse, 17R-RvD1 administrations reduced leukocyte recruitment, synovitis, paw edema, pannus intrusion, cartilage damage, and clinical score [113]. In contrast, RvE1 did not affect the incidence or severity of collagen-induced arthritis in mice, measured as synovial inflammation, PG depletion, chondrocyte death, and cartilage/bone erosion [153]. Neither did it influence the release of pro-inflammatory cytokines from human RA synovial tissue. However, RvD1 and RvE1 reduced osteoclast differentiation and bone resorption in vitro [158, 159]. In addition, RvD1 decreased paw inflammation and cartilage and bone destruction in arthritic mice [159].

Regarding research on arthritis-related pain, SF RvE2 levels were inversely associated with pain score in patients with different types of arthritis [169]. Administrations of RvE1 and RvD1 attenuated inflammatory pain in mouse models without affecting the basal pain perception [170]. 17-HDHA and its product, aspirin-triggered RvD1, displayed anti-hyperalgesic properties in rats with complete Freund᾽s adjuvant-induced arthritis [171]. The practical usefulness of SPM in the treatment of RA pain remains to be elucidated in the future.

To sum up, SPM have significant treatment potential for different kinds of arthritis. As these molecules are unstable, synthetic analogs are being developed for therapeutic purposes. SPM could have several benefits, as they reduce pain perception and display chondro- and bone-sparing properties without provoking immunosuppression or tolerance.

## Conclusions and Research Agenda for Future Studies

Some general patterns that emerge from the collected literature can be summarized as follows:

- There exists a striking discordance between the large body of literature on the adverse and beneficial effects of individual FA on joint health and the lack of effective treatments for joint destruction in OA and RA.
- Dietary interventions can provide some alleviation of symptoms and pain in RA, but high-quality randomized controlled trials are necessary to investigate the effects of dietary FA supplements on arthropathies.
- Existing studies only concentrate on a few FA, and there is a lack of research on especially 20–24C MUFA. Distinct anatomical locations—hip and shoulder in addition to the usually studied knee—should be compared to reveal whether there are differences in their lipid manifestations in joint diseases.
- It would be of utmost importance to develop biomarkers for early disease detection and for monitoring of progress, but this has been difficult regarding lipidology. It is possible that lipids have more promise in the development of therapeutic interventions than as biomarkers.
- SPM have potential in the pain relief and treatment of joint diseases, and especially RvD1, RvE1, and LXA_4_ are promising for translational research.
- EV are an emerging research subject that is most attractive regarding translational medicine as they have potential to repair damaged cartilage in animal models. In addition to bioactive lipids carried by EV, the FA composition of EV membrane and its effects on target tissues should be a focus of future studies.

## Data Availability

Not applicable

## Abbreviations

ADAMTS: a disintegrin and metalloproteinase with thrombospondin motifs
CLA: conjugated linoleic acid
COX: cyclooxygenase
DHET: dihydroxyeicosatrienoic acid
15d-PGJ_2_: 15-deoxy-∆^12,14^-prostaglandin J_2_
ECM: extracellular matrix
EV: extracellular vesicle
FA: fatty acid
FLS: fibroblast-like synoviocyte
GAG: glycosaminoglycan
HDHA: hydroxydocosahexaenoic acid
HEPE: hydroxyeicosapentaenoic acid
HETE: hydroxyeicosatetraenoic acid
HODE: hydroxyoctadecadienoic acid
IFP: infrapatellar fat pad
IL: interleukin
iNOS: inducible nitric oxide synthase
LOX: lipoxygenase
LPS: lipopolysaccharide
LTB_4_: leukotriene B_4_
LXA_4_: lipoxin A_4_
MaR: maresin
MMP: matrix metalloproteinase
MUFA: monounsaturated fatty acid
NO: nitric oxide
NSAID: nonsteroidal anti-inflammatory drug
OA: osteoarthritis
PD1: protectin D1
PDX: protectin DX
PG: proteoglycan
PGD_2_: prostaglandin D_2_
PGE_2_: prostaglandin E_2_
PGF_2*α*_: prostaglandin F_2*α*_
PL: phospholipid
PUFA: polyunsaturated fatty acid
RA: rheumatoid arthritis
RvD: D-series resolvin
RvE: E-series resolvin
SF: synovial fluid
SFA: saturated fatty acid
SPM: specialized pro-resolving lipid mediator
TIMP: tissue inhibitor of metalloproteinases
TXB_2_: thromboxane B_2_
